# Causal relationship between tea intake and cardiovascular diseases: A Mendelian randomization study

**DOI:** 10.3389/fnut.2022.938201

**Published:** 2022-09-26

**Authors:** Ning Gao, Ming Ni, Jiangwei Song, Minjian Kong, Dongdong Wei, Aiqiang Dong

**Affiliations:** Department of Cardiovascular Surgery, The Second Affiliated Hospital of Zhejiang University School of Medicine, Hangzhou, China

**Keywords:** cardiovascular disease, tea intake, Mendelian randomization, genetics, causal correlation

## Abstract

**Background:**

Although studies suggest that tea consumption is associated with a reduced risk of cardiovascular disease (CVD). There is no unified conclusion about the potential relationship between tea drinking and CVD. We used a two-sample Mendelian randomized (MR) analysis to systematically explore the causal relationship between tea intake and CVD subtypes for the first time. Furthermore the mediating effect of hypertension was also explored by a two-step MR.

**Methods:**

Genetic instruments for tea intake were identified from a genome-wide association studies (GWAS) involving 447,485 people. Summary data on cardio-vascular disease came from different GWAS meta-analysis studies. In the first step we explored the causal effect of tea intake and CVD. In the second step, we examined the association of hypertension with heart failure and ischemic stroke and estimated the mediating effect of hypertension. Inverse variance weighted MR analysis was used as the primary method for causal analysis. A further sensitivity analysis was performed to ensure robustness of the results.

**Results:**

One standard deviation increase in tea intake was associated with a 25% (OR = 0.75, 95%CI = 0.61–0.91, *p* = 0.003) lower risk of hypertension, a 28% (OR = 0.72, 95%CI = 0.58–0.89, *p* = 0.002) lower risk of heart failure, and a 29% (OR = 0.71, 95%CI = 0.55–0.92, *p* = 0.008) lower risk of ischemic stroke, respectively. And the association between tea drinking and the risk of heart failure and ischemic stroke may be mediated by hypertension. Sensitivity analyses found little evidence of pleiotropy.

**Conclusion:**

Our two-sample MR analysis provided genetic evidence that tea intake was significantly associated with a reduced risk of hypertension, heart failure, and ischemic stroke, and that hypertension may be a potential mediator. Further large randomized controlled trials should be conducted to confirm the causal effect of tea consumption on cardiovascular disease risk.

## Introduction

Cardiovascular disease (CVD) is the result of complications in the heart and blood vessels. Globally, CVD remains a rising global epidemic, with more than 17 million deaths due to CVD annually, according to world Health Organization estimates (1). And it is expected to rise to 23.6 million by 2030 (2). Due to the heavy social and family burden of CVD, early intervention and prevention strategies are particularly important (3). Recently, the intervention of dietary factors in CVD has attracted attention because it is often easily accepted (4).

Tea is the second most consumed beverage in the world and contains a variety of bioactive components (5). Tea is a major source of flavonoid intake in humans, particularly flavane-3-ols, which have been experimentally shown to prevent or delay atherosclerosis (6, 7). Flavonoids have also been shown to have antithrombotic and anti-inflammatory properties. As a result, it is thought that the risk of CVD can be reduced by changing tea intake (8, 9). A retrospective study based on 612 patients suggested that greater green tea consumption was significantly inversely associated with coronary heart disease (CHD) prevalence (10). However, another study involving 9253 patients found that tea consumption and the risk with myocardial infarction (MI) was not evident (11). A case-control study found that green tea intake presented as a protective factor against the incidence of atrial fibrillation (AF) (OR = 0.349, 95%CI = 0.253–0.483, *p* < 0.001) (12). There are also cohort studies showing no correlation between tea drinking and AF (13). Another study noted that habitual tea drinkers (≥3 times a week for at least 6 months) had a 14% [hazard ratio (HR) = 0.86, 95%CI = 0.80–0.91] lower risk of developing hypertension compared with non-habitual tea drinkers (14). Increased caffeine intake (coffee/black tea) was significantly associated with decreased heart failure (HF) (15). In addition, a prospective cohort study of 365682 participants (ages 50–74) from the UK Biobank found that drinking 2–3 cups of tea a day was associated with a 32% lower risk of stroke (HR = 0.68, 95%CI = 0.59–0.79, *p* < 0.001) compared with those who did not drink tea or coffee (16). However, these studies have a high risk of confounding factors and reverse causality. Therefore, it is unclear whether there is a potential causal relationship between tea consumption and CVD.

Mendelian randomization (MR) is another method to account for observational bias (17), which uses genetic variation to estimate a causal relationship between exposure and outcome. Relying on the random assignment of genetic variation during meiosis, MR mimics the natural “random trials” in a population (18). It is crucial that genetic variation is generally free from confounding factors, while the risk of reverse causation is minimized (19). Thus, MR provides a robust understanding of the causal relationship between exposure and outcome. In addition, a two-step MR is an effective way to explore mediating effects (20).

## Materials and methods

### Study design

**FIGURE 1:**
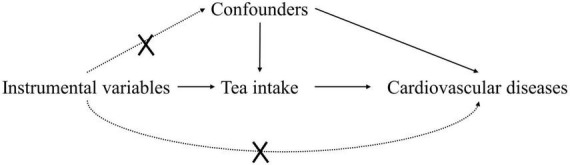
Study design flowchart of the Mendelian randomization study.

This study is a two-sample MR study based on genetic data obtained from genome-wide association studies (GWAS), and we followed the latest guidelines for MR analysis (21). This study was conducted under three basic assumptions: (1) The genetic variants are closely associated with tea intake; (2) The genetic variation is not associated with any potential confounders; (3) the genetic variants are not associated with CVD except via the way of tea intake. In addition, other assumptions should be satisfied, including linearity and no statistical interaction (22). A conceptual schematic of current MR research was shown in Figure 1.

**FIGURE 2:**
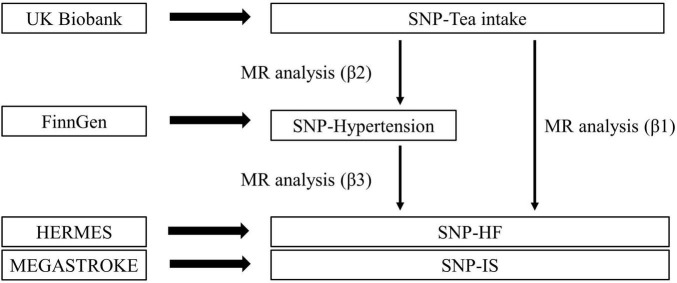
Overview of the two-step method Mendelian randomization. Total effect= β1; Mediation effect= β2*β3; Direct effect= β1–β2*β3; Proportion mediated= (β2*β3)/β1.

Based on the two-sample MR analysis, if an association exists between tea intake and hypertension, two-step MR analysis will be performed to explore possible mediating effects (23). In the first step, we verified the effect (β2) of tea intake on mediators, and in the second step, we estimated the effect (β3) of mediators on CVD (Figure 2).

The data used in this study were summary-level data, so all informed consent and ethical approval were obtained in the original study.

### Instrumental variable selection

Genetic instrumental variables for tea intake were extracted from the UK Biobank (UKB) involving 447,485 participants (Phenotype Code:1488_raw) (GWAS ID: ukb-b-6066). This GWAS adjusted for sex, genotyping arrays, and the top 10 principal components, and considered correlations and stratification. Data on habitual tea drinking was obtained through a questionnaire, which involved the question: “How many cups of tea (including black and green tea) do you drink per day?” According to the survey, the mean value of tea intake is 3.51 and the standard deviation (SD) is 2.85. Specific information can be found in MRC-IEU^1^. All genetic variants significantly associated with tea intake were selected as instrumental variables (IVs) (*p* < 5 × 10^–8^). A total of 41 SNPs reached GWAS. The association of these SNPs with tea intake may be due to three mechanisms. First, these SNPs may be associated with taste loci. It has been shown that as a bitter beverage, tea intake is associated with bitterness-associated loci. Secondly, these SNPs are associated with caffeine metabolism and caffeine action targets. Thus regulating the concentration of bioactive components may indirectly influence tea drinking behavior (24). In addition, the possible association between SNPs and olfactory receptors (25), and individuals who prefer floral scents may be more inclined to drink tea.

These single nucleotide polymorphisms (SNPs) were clumped based on the linkage disequilibrium, defined by *r*^2^ < 0.001 and clumping window >10,000 kb (26). It was critical to exclude potential horizontal pleiotropy, SNPs associated with confounders or risk factors for the outcome (diabetes, lipids, body mass index, and smoking) were excluded (threshold of *p*-value = 1E-5, *r*^2^ = 0.8)^2^ (27). In addition, we performed a strength assessment of IVs. The *F*-statistic and *R*^2^ (the proportion of variance explained) were introduced in this process (28, 29). If *F* is greater than 10, the study is less likely to be affected by weak instrumental bias.

Hypertension is considered as a possible mediating factor. Therefore, in this study, if there is a causal association between tea intake and hypertension, the same criteria will be implemented to extract IVs significantly associated with hypertension for a two-step MR analysis.

### Cardiovascular disease data sources

We used AF GWAS summary-statistics from a large meta-analysis data. The GWAS compared six studies (Nord-Trøndelag Health Study (HUNT), AFGen Consortium, deCODE, Michigan Genomics Initiative (MGI), UK Biobank and DiscovEHR), including 60,620 cases and 970,216 controls (30). Summary statistic data for CHD was from the Coronary Artery Disease Genomewide Replication and Meta-analysis plus the Coronary Artery Disease Genetics (CARDIoGRAMplusC4D), involving 60,801 cases and 123,504 controls from 48 cohorts (about 77% of participants of European ancestry). Summary-level data for hypertension was extracted from the FinnGen^3^. A total of 55,917 cases and 162,875 controls were included in this GWAS. We used the latest GWAS meta-analysis of HF, involving 60,620 patients and 970,216 controls in 26 studies (31). For ischemic stroke (IS), summary-data was extracted from the GWAS of MEGASTROKE consortium, involving 40,585 cases and 406,111 controls (32). Information on all genetic datasets in this study was shown in Table 1 and Supplementary Table 1.

**TABLE 1:** Data sources and instrumental variables strength assessment.

Trait	Data sources	Sample size (case/control)	Ancestry	*R*^2^ (total)	*F*- statistic (total)
**Exposure**					
Tea intake	UK Biobank (MRC-IEU)	447,485	European		
**Outcome**					
Atrial fibrillation	HUNT, UK Biobank, deCODE, DiscovEHR, MGI and AFGen	60,620/970,216	European	0.17	21.35
Coronary heart disease	CARDIoGRAMplusC4D	60,801/123,504	77% European	0.17	21.44
Hypertension	FinnGen	55,917/162,875	European	0.17	21.79
Heart failure	HERMES	47,309/930,014	European	0.11	18.15
Ischemic stroke	MEGASTROKE	40,585/406,111	European	0.13	16.86

CARDIoGRAMplusC4D, Coronary Artery Disease Genome-wide Replication and Meta-analysis plus The Coronary Artery Disease Genetics; GENEVA, Gene Environment-Association Studies; WTCCC, Wellcome Trust Case Control Consortium; FUSION, Finland–United States Investigation of NIDDM Genetics; GERA, Resource for Genetic Epidemiology Research on Aging; NuGENE, Northwestern NuGENE project; HUNT, The Nord-Trøndelag Health Study; MGI, the Michigan Genomics Initiative; deCODE, the Collaborative Analysis of Diagnostic Criteria in Europe study; F = R^2^(N-K-1)/[K(1–R^2^)], R^2^ = 2 × (1–EAF) × EAF × (β/SD)^2^, SD = SE × N^1/2^, where EAF is the effect allele frequency, β is the estimated effect on tea intake, N is the sample size of the GWAS and SE is the standard error of the estimated effect.

### Statistical analysis

The Wald ratio was used to estimate the effect of exposure on the outcome for each IV (33), and then the inverse variance weighted (IVW) meta-analysis of each Wald ratio was performed to obtain MR estimates (34). In addition, MR-Egger (35), median weighting (36), Maximum-likelihood (37), MR-robust adjusted profile score (MR-RAPS) (38) and MR-pleiotropy residual sum and outlier (MR-PRESSO) (39) were used as a complement to IVW. Each statistical method makes different assumptions about IVs. In this study, the fixed-effects model IVW method was used as the main statistical method (40). The details of the analysis method are shown in Supplementary Table 2.

The two-step MR analysis was performed to estimate mediating effects. In this study, the total effect (β1) was the effect of tea intake on CVD. And the magnitude of the direct and indirect effects could be estimated separately by this method (Figure 2). This mediated analysis method is based on the premise that the MR analyses in all steps are statistically significant, otherwise it will not be performed.

### Sensitivity analyses

Various approaches were used for sensitivity analysis. Firstly, The value of Cochrane’s Q was applied to assess the heterogeneity (41). If the *p*-value of Cochran’s Q was less than 0.05, the IVW method with multiplicative random-effects model was used as the primary outcome; otherwise, the fixed-effects model was applied (42). Second, in order to avoid assumptions 2 and 3, we used MR-Egger intercept to evaluate horizontal pleiotropy (35). If the *p*-value of the MR-Egger intercept was less than 0.05, the instrumental variable was considered to be heavily influenced by horizontal pleiotropy, and this result was unreliable. Meanwhile, funnel plots were also used to examine horizontal pleiotropy. Third, the MR-PRESSO was performed to automatically detect and correct outliers to validate the results of the IVW method (39). In addition, the forest plot reflected the association of exposure with outcome in each SNP.

The mRnd was used to calculate the statistical power^4^. All the data analyses were performed using R (Version 4.1.2), and R package “TwoSampleMR,” “MR-PRESSO,” and “mr. raps.”

## Results

Detailed characterizations of all SNPs involved in the current study are shown in Supplementary Tables 3–9. We excluded SNPs associated with CVD or its confounders (rs10741694, rs9937354, rs4410790), and also excluded palindromic SNPs.

### Mendelian randomized analysis of tea intake on cardiovascular disease risk

**FIGURE 3:**
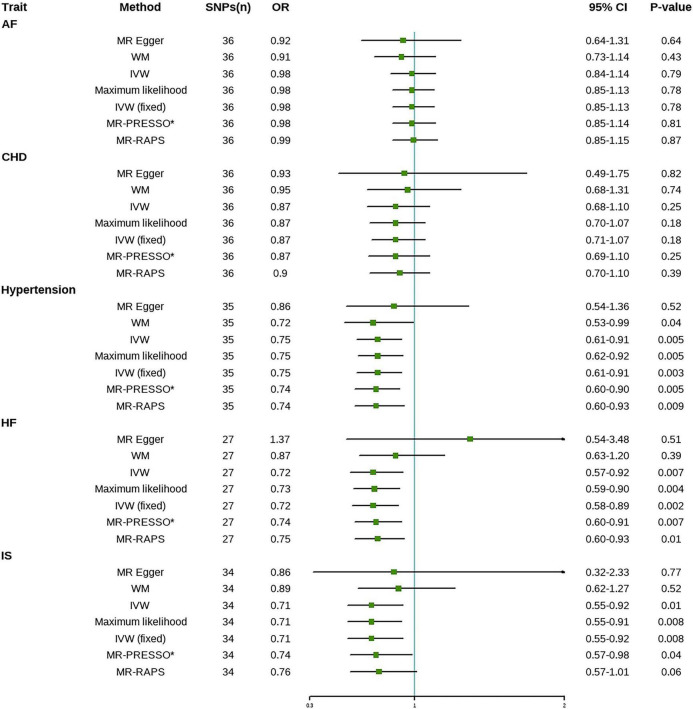
Mendelian randomization estimates of tea intake on the risk for CVD. AF, atrial fibrillation; CHD, coronary heart disease; HF, heart failure; IS, ischemic stroke; SNPs, Single nucleotide polymorphisms; OR, Odds ratio; CI, Confidence interval; IVW, inverse-variance weighted; IVW (fixed), fixed-effects inverse-variance weighted; MR-RAPS, MR-robust adjusted profile score; MR-PRESSO, MR-pleiotropy residual sum and outlier; *No outlier was detected.

Figure 3 reports MR estimates of tea intake on CVD risk. In IVW MR analysis, one SD increase in tea intake was associated with a 25% (OR = 0.75, 95% CI = 0.61–0.91, *p* = 0.003) lower risk of hypertension, a 28% (OR = 0.72, 95% CI = 0.58–0.89, *p* = 0.002) lower risk of HF, and a 29% (OR = 0.71, 95% CI = 0.55–0.92, *p* = 0.008) lower risk of IS, respectively. However, IVW estimates showed that genetically predicted tea intake was not significantly associated with the risk of AF (OR = 0.98, 95% CI = 0.85–1.13, *p* = 0.78) and CHD (OR = 0.87, 95% CI = 0.71–1.07, *p* = 0.18). Most statistical models were directionally consistent with the IVW method. No outliers were found in the MR-PRESSO analysis.

No evidence of heterogeneity was found in any of the analyses in this study (Table 2). Therefore, the fixed-effects model IVW method was used as the primary method of this study. The *p*-values of the MR-Egger intercepts for all CVD subtypes were greater than 0.05, indicating that there was no horizontal pleiotropy in the analysis results, which satisfied assumptions 2 and 3. Furthermore, the funnel plot was symmetric (Supplementary Figure 1), and the leave-one-out method suggested that the association between tea intake and CVD risk was not driven by a single SNP (Supplementary Figure 2). The forest plot was shown in Supplementary Figure 3. The F-statistic for each study was greater than the empirical threshold of 10, indicating the validity of IVs (Table 1). The statistical power was 100% for all outcomes except for atrial fibrillation where the statistical power was 53% (Supplementary Table 10). Therefore, the effect of tea intake on AF may be undetectable due to the lower statistical power.

**TABLE 2:** Pleiotropy and heterogeneity test of the tea intake from CVD GWAS.

Outcomes	Pleiotropy test			Heterogeneity test					
	MR-Egger			MR-Egger			Inverse-variance weighted		
	Intercept	SE	*p*	Q	Q_df	Q_*p*val	Q	Q_df	Q_*p*val
Atrial fibrillation	0.001	0.003	0.70	39.16	34	0.25	39.34	35	0.28
Coronary heart disease	−0.001	0.006	0.82	47.70	34	0.06	47.77	35	0.07
Hypertension	−0.003	0.004	0.51	37.03	33	0.29	37.52	34	0.31
Heart failure	−0.011	0.008	0.17	30.64	25	0.20	33.04	26	0.16
Ischemic stroke	−0.003	0.008	0.70	35.70	32	0.30	35.86	33	0.34

df, degree of freedom; MR, Mendelian randomization; Q, heterogeneity statistic Q.

### Mendelian randomized analysis of hypertension on heart failure and ischemic stroke

Table 3 reports MR estimates of hypertension on HF and IS. The IVW method showed that hypertension was positively associated with a higher risk of HF (OR = 1.21, 95% CI = 1.15–1.28, *p* = 7.37E-12) and IS (OR = 1.32, 95% CI = 1.24–1.41, *p* = 5.80E-19). Median weighting, Maximum-likelihood and MR-PRESSO were directionally consistent with the IVW method. The MR-PRESSO method detected outliers, but the significance of the results in removing the outliers did not disappear, so this result is reliable.

**TABLE 3:** Mendelian randomization estimates of hypertension on the risk for HF and IS.

Trait	Method	SNPs (*n*)	OR	95% CI	*P*-value	*P*-value for Cochran’s Q	*P*-value for Mr-egger intercept	*F*- statistic (total)
Heart failure							0.10	18
	MR Egger	47	1.04	0.87–1.25	0.64	6.50E-06		
	Weighted median	47	1.18	1.12–1.25	8.50E-09			
	IVW	47	1.21	1.15–1.28	7.37E-12	1.61E-06		
	Maximum likelihood	47	1.22	1.18–1.27	2.30E-25			
	IVW (fixed)	47	1.21	1.17–1.26	4.01E-25			
	MR-PRESSO*	47	1.21	1.15–1.27	1.59E-09			
Ischemic stroke							0.12	17
	MR Egger	53	1.12	0.91–1.38	0.28	1.31E-04		
	Weighted median	53	1.24	1.15–1.34	6.36E-08			
	IVW	53	1.32	1.24–1.41	5.80E-19	5.33E-05		
	Maximum likelihood	53	1.34	1.28–1.40	8.50E-35			
	IVW (fixed)	53	1.32	1.26–1.38	2.42E-35			
	MR-PRESSO*	53	1.35	1.26–1.44	1.20E-12			

SNPs, Single nucleotide polymorphisms; OR, Odds ratio; CI, Confidence interval; IVW, inverse-variance weighted; IVW (fixed), fixed-effects inverse-variance weighted; MR-RAPS, MR-robust adjusted profile score; MR-PRESSO, MR-pleiotropy residual sum and outlier.

*No outlier was detected.

Cochran’s *Q*-test confirmed the presence of heterogeneity. Therefore, the causal estimation of hypertension on HF and IS was performed using the random-effects IVW method as the main method. The funnel plot was symmetric (Supplementary Figure 4). In addition, we found no evidence of the presence of potential horizontal pleiotropy and the MR findings were not driven by any single SNP (Supplementary Figure 5). The forest plot was shown in Supplementary Figure 6. The F-statistic for each study was greater than the empirical threshold of 10, indicating the validity of IVs (Table 3). The statistical power for both HF and IS outcomes was greater than 80% (Supplementary Table 10).

### Mediating effects of hypertension traits on the association of tea intake with cardiovascular disease risk

We used the two-step MR to explore whether the association between tea drinking and the risk of HF and IS were mediated through hypertension. Our results indicated that the proportion mediated of the mediating effect of hypertension was 17% (OR = 0.95) in the association between tea and the risk of HF and 24% (OR = 0.92) in the association between tea and the risk of IS (Table 4).

**TABLE 4:** Mediated Mendelian randomization analysis.

Trait	Total effect	Direct effect	Mediation effect	Proportion mediated (%)
Heart failure	0.72	0.76	0.95	17
Ischemic stroke	0.71	0.77	0.92	24

## Discussion

This study used summary-level data from the large GWAS to explore the causal relationship between tea intake and CVD. Our MR study found that increased tea intake may reduce the risk of hypertension, HF and IS. Meantime, this study revealed that the reduced risk of HF and IS may be mediated by the lower risk of hypertension. In addition, tea intake may not be associated with the risk of CHD and AF. However, due to the relatively low statistical power of tea intake on atrial fibrillation, a true cause-and-effect relationship may not be detected.

Previous observational studies provided inconsistent results in the association between tea intake and CVD risk. An observational study showed that consumption of 1 dL of tea per day was associated with lower diastolic blood pressure and pulse pressure, while no association was observed between blood pressure and coffee consumption (43). A small clinical trial found that drinking two cups of green tea a day for 14 days improved blood pressure (44). Likewise, a large cohort study showed that tea consumption was associated with a reduced risk of major coronary events (45). However, some prospective studies have not found a statistically significant association between tea consumption and coronary artery disease (46, 47). A cohort study of 128,280 adults showed that tea was associated with a lower risk of ischemic and hemorrhagic stroke (48). However, An epidemiological study showed a lack of association between tea and CVD, either CHD or IS (49). Potential confounders and reverse causality may have contributed to inconsistent results from observational studies. In addition, as a traditional risk factor, hypertension tends to increase the prevalence and mortality of HF and IS. An observational study suggests that sustained blood pressure control may be beneficial for stroke and HF outcomes (50). Similarly, a MR analysis suggests that genetic susceptibility to hypertension is associated with a higher risk of IS (51), which is consistent with our findings.

Some potential mechanisms were reported. A cup of tea contains 35–55 mg of caffeine, which may reduce cardiovascular risk by increasing endothelial nitric oxide release leading to vasodilation, as well as its antioxidant properties (52). Polyphenolic compounds contained in tea inhibit myocardial fibrosis, oxidative stress, inflammation and cardiomyocyte apoptosis (53). However, an MR study showed no association between caffeine and CVD (54). Polyphenolic compounds in tea are also thought to have possible cardiovascular benefits. Several studies have shown that epigallocatechin-3-gallate (EGCG) can stimulate the proliferation and migration of vascular endothelial cells (55) and inhibit the proliferation of vascular smooth muscle cells induced by homocysteine (56), thus protecting endothelial cells. EGCG also plays an important role in relieving inflammation (57) and reducing oxidative stress (58). HF and IS often occur with the involvement of ischemia/reperfusion injury, and EGCG may mitigate this injury and thus reduce the risk of disease (59). EGCG modulates blood pressure in hypertensive rats by increasing nitric oxide (NO) concentrations (60), and it also modulates the expression of endothelin-1 (ET-1), which is a very potent vasoconstrictor (61). Moreover, a class of polyphenolic compounds called flavonoids plays an important role in the treatment and control of hypertension (62). These mechanisms may partly explain the association of tea with a lower risk of CVD.

Our study includes several advantages. First, MR analysis was performed for the first time to explore the potential causal relationship between tea intake and a range of CVD, which largely overcomes the limitations of traditional observational studies (environmental confounders, reverse causality, insufficient sample size). Second, the use of summary-level data, with sufficient numbers of cases, greatly improved the statistical power to detect causal effects. Third, all studies had genomic controls, suggesting that our results are unlikely to be affected by genome inflation. Fourth, we performed a mediation analysis using two-step MR and showed that the association of tea intake with HF and IS may be mediated by hypertension. In addition, sensitivity analyses and repeated analyses of multiple statistical models explained different patterns of pleiotropy, strengthening the evidence for our findings.

This study strictly satisfies three assumptions. For assumption 1, all SNPs selected as IVs reached GWAS and their minor allele frequencies were >0.01. Further, we removed the linkage disequilibrium of the IVs. In addition, the F-statistic values were all >10. For assumption 2, we searched for secondary phenotypes of each SNP and excluded SNPs associated with CVD and its risk factors. For assumption 3, all SNPs significantly associated with outcomes were not included in this study. Meanwhile the results of MR-Egger intercept test supported assumption 3.

The study also has some limitations. Firstly, the participants involved in this study were primarily of European ancestry, which limits the applicability of the results to other populations. Secondly, summary-level data limited us to non-linear MR studies and subgroup analyses. Thirdly, although we tried our best to avoid sample overlap, the samples from the HF and AF datasets contain a fraction of UKB data, which may lead to slightly biased MR estimates. However, no other GWAS that were available and included a sufficient number of cases were reported. Furthermore, given the low statistical power, we did not explore specific stroke subtypes. Further MR studies will be valuable when a larger sample size of GWAS data is available. Finally, it was difficult to demonstrate that the results of the analysis were completely unaffected by horizontal pleiotropy. In this regard, we applied multiple sensitivity analyses and found no evidence of potential pleiotropy. Therefore, we consider the analytical results to be sufficiently reliable.

## Conclusion

In summary, our two-sample MR analysis provides genetic evidence that tea intake is significantly associated with the decreased risk of hypertension, heart failure, and ischemic stroke. Also, we found that the association between tea intake and the risk of heart failure and ischemic stroke may have been mediated by hypertension. Next steps should include further MR studies to investigate the association of tea intake with other CVD subtypes. Non-linear MR studies at higher levels of tea consumption should also be investigated, which will better define the potential role of tea in preventing the onset and progression of CVD. Randomized controlled trials of tea-based interventions should also be on the agenda.

## Data availability statement

The original contributions presented in this study are included in the article/Supplementary material, further inquiries can be directed to the corresponding author/s.

## Author contributions

NG, MN, and AD designed the study and drafted the article. JS and MK conducted data acquisition. NG, MK, MN, DW, and AD performed data analysis and manuscript revision. All authors read and approved the final manuscript.

## Footnotes

1
https://data.bris.ac.uk/data/

2
http://www.phenoscanner.medschl.cam.ac.uk/

3
https://www.finngen.fi/fi

4
https://cnsgenomics.shinyapps.io/mRnd/
