# Supplementary material for: Causal relationship between tea intake and cardiovascular diseases: A Mendelian randomization study
Source: Front Nutr. 2022 Sep 26;9:938201. doi: 10.3389/fnut.2022.938201 (PMC9548982; doi:10.3389/fnut.2022.938201)
Supplement: Supplementary file 1 [file Data_Sheet_1.docx]

Supplementary Material

## Supplementary Figures


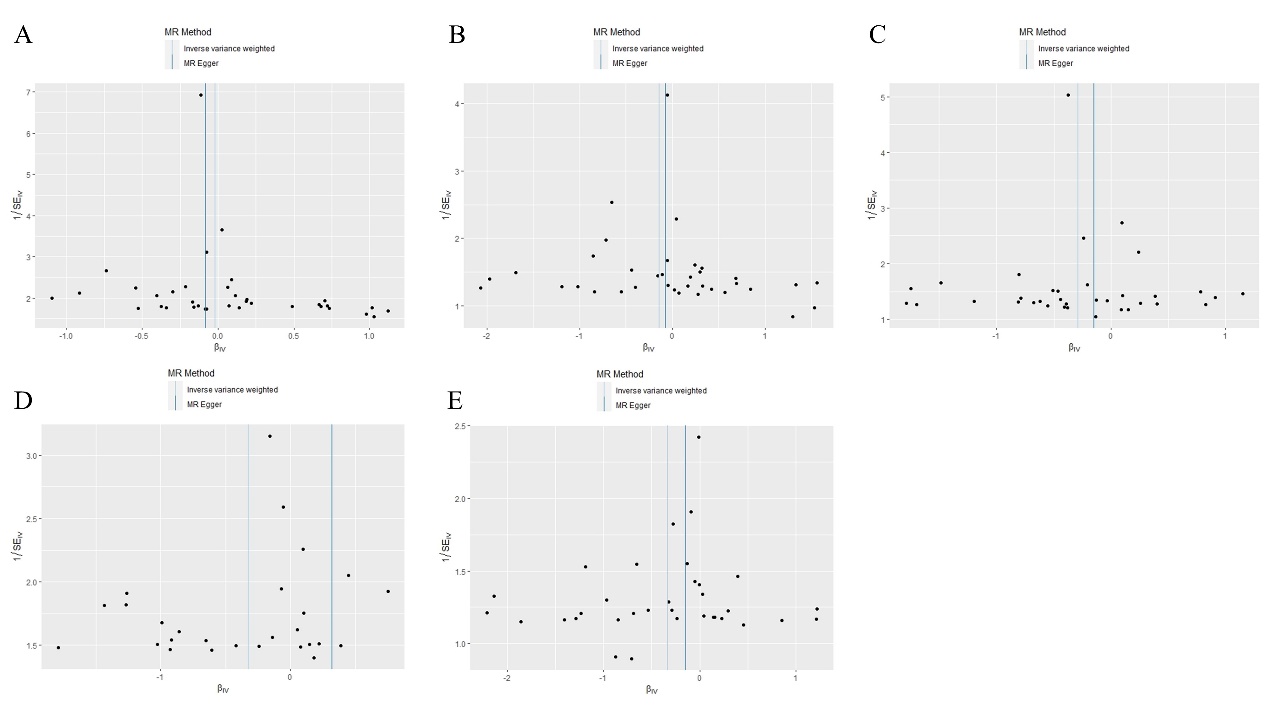


Figure S1 Funnel plot: (A) tea intake and atrial fibrillation; (B) tea intake and coronary heart disease; (C) tea intake and hypertension; (D) tea intake and heart failure; (E) tea intake and ischemic stroke.


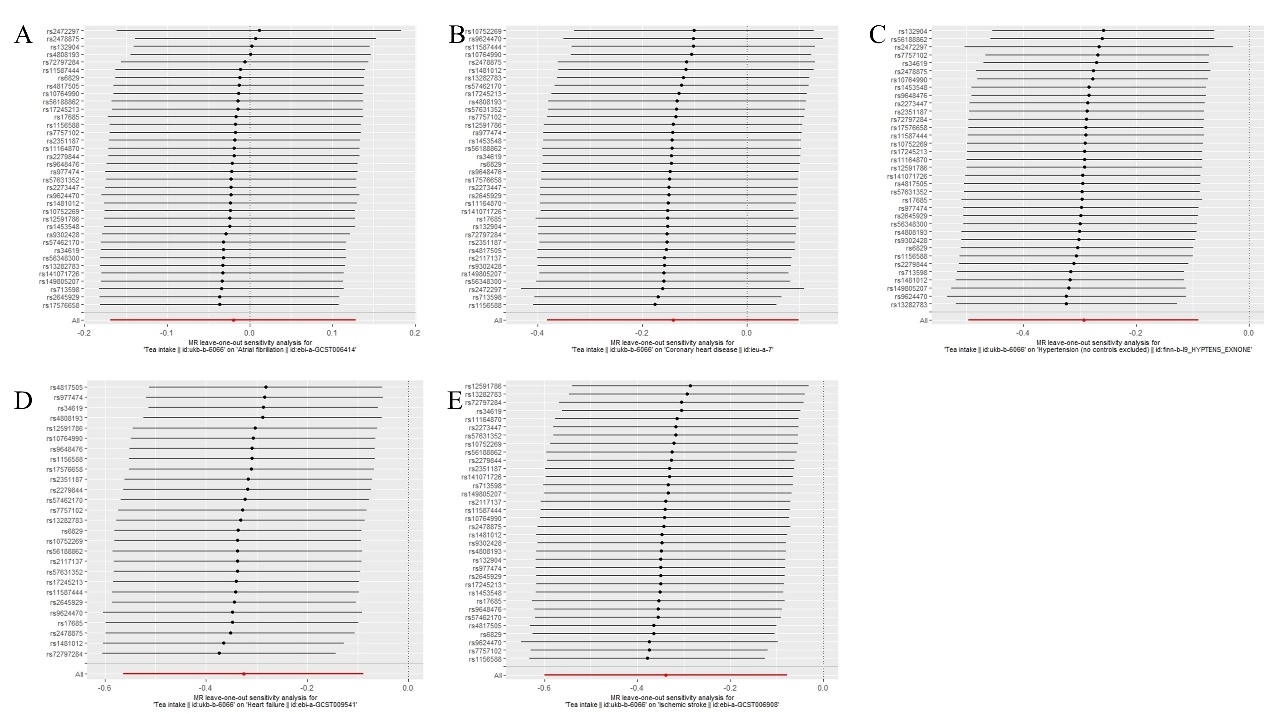


Figure S2 Leave-one-out method: (A) tea intake and atrial fibrillation; (B) tea intake and coronary heart disease; (C) tea intake and hypertension; (D) tea intake and heart failure; (E) tea intake and ischemic stroke.


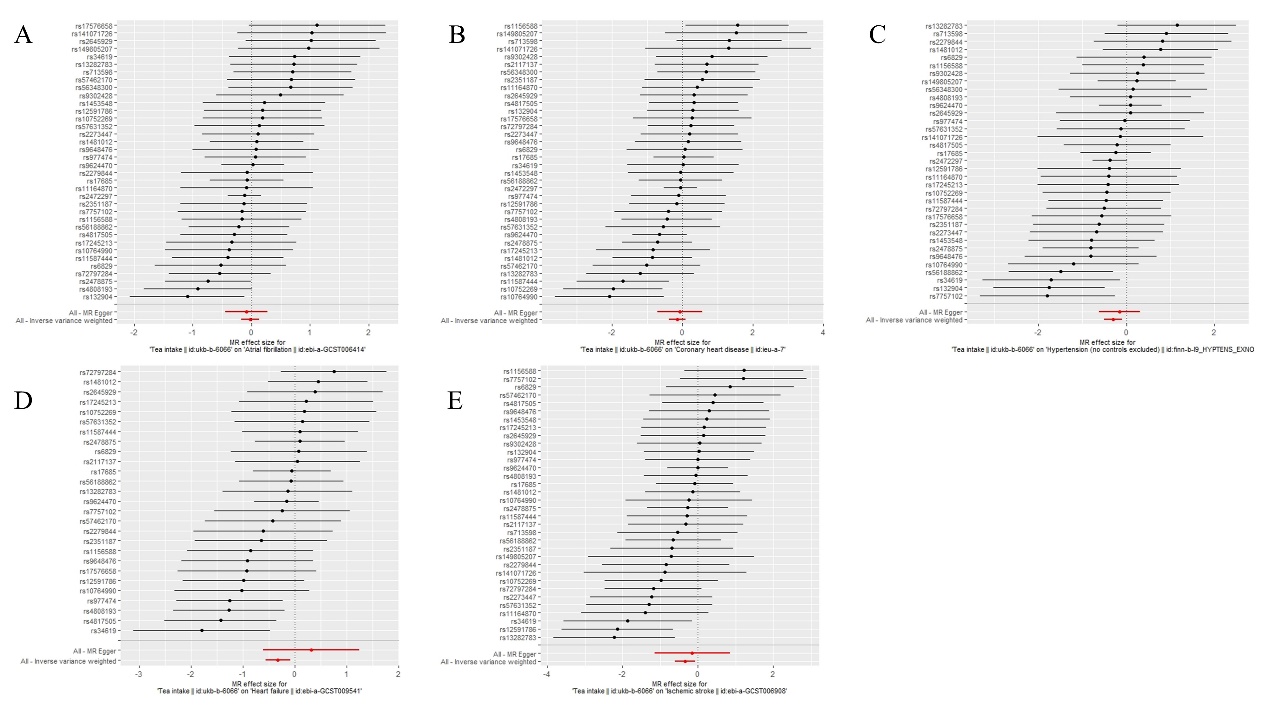


Figure S3 Forest plot: (A) tea intake and atrial fibrillation; (B) tea intake and coronary heart disease; (C) tea intake and hypertension; (D) tea intake and heart failure; (E) tea intake and ischemic stroke.


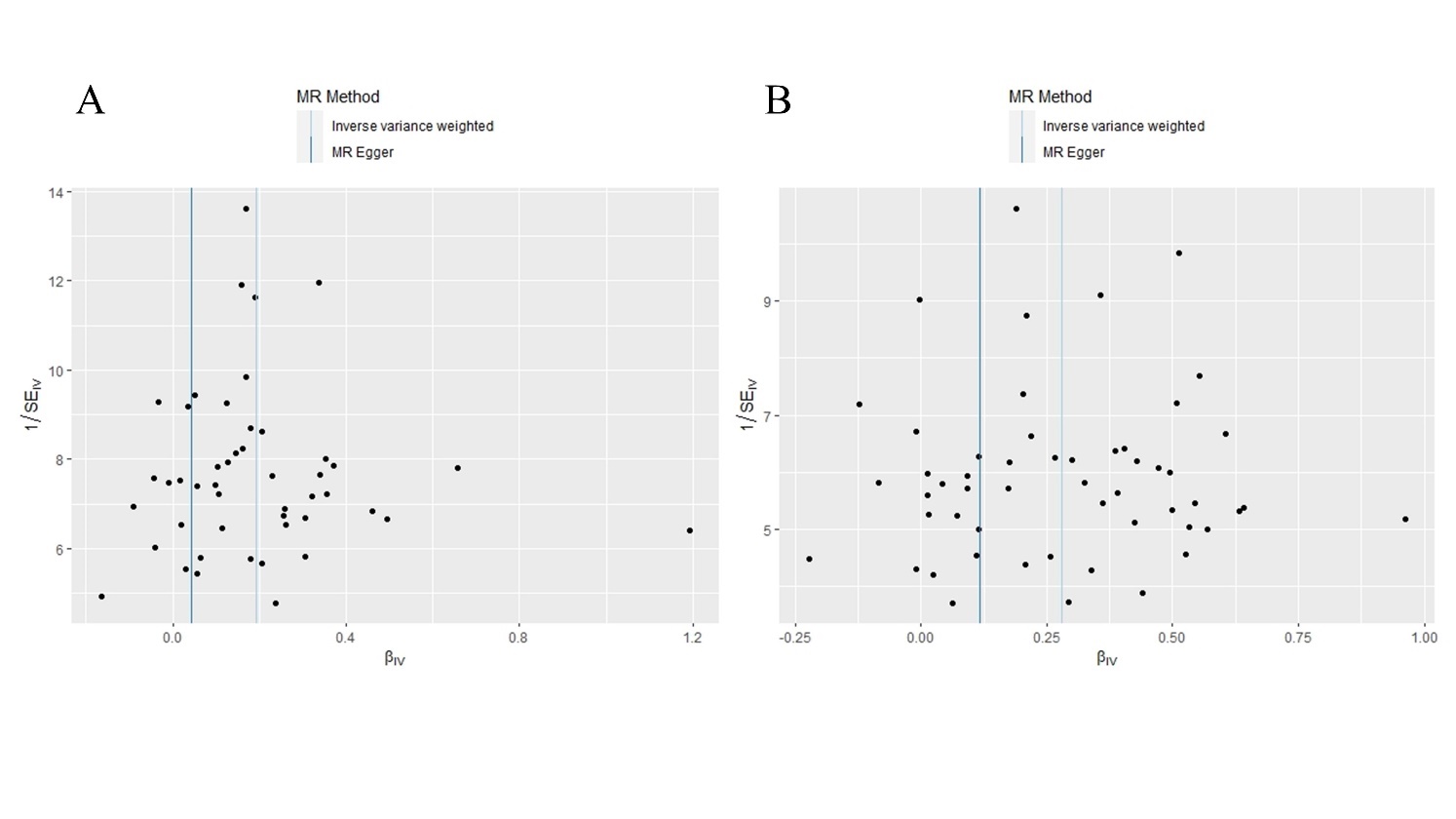


Figure S 4 Funnel plot: (A) hypertension and heart failure; (E) hypertension and ischemic stroke.


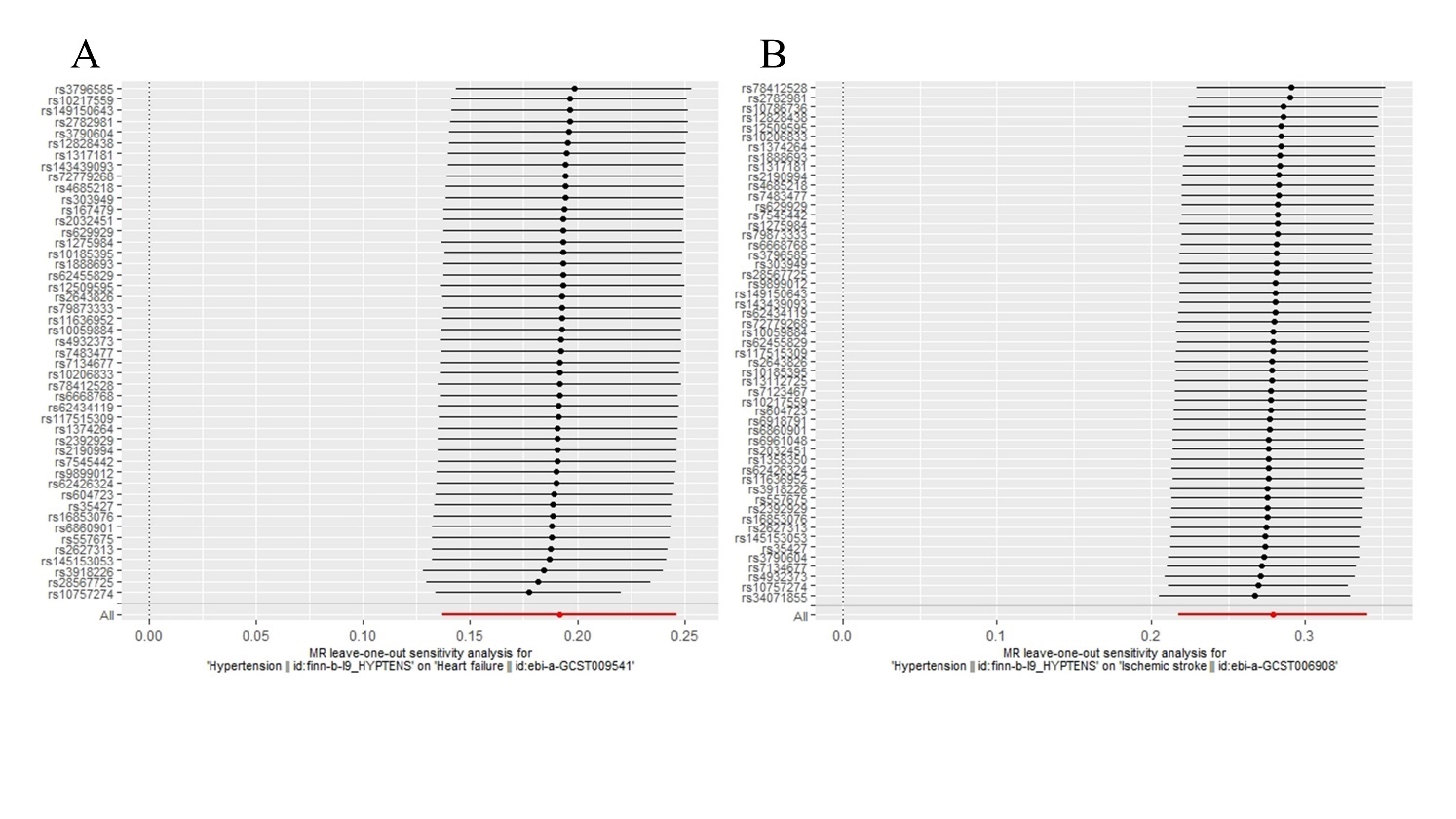


Figure S5 Leave-one-out method: (A) hypertension and heart failure; (E) hypertension and ischemic stroke.


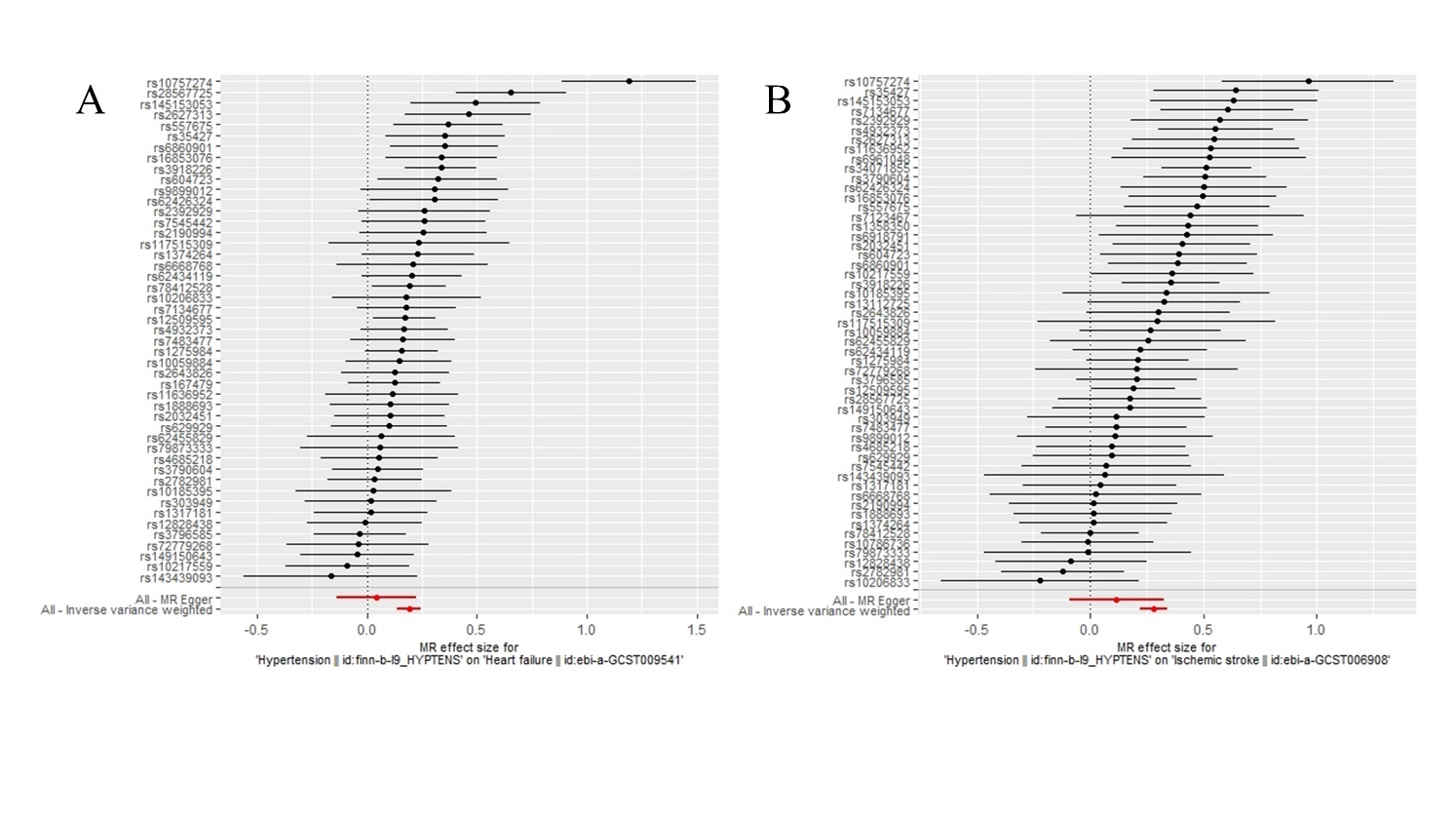


Figure S 6 Forest plot: (A) hypertension and heart failure; (E) hypertension and ischemic stroke.

Table S1 Characteristics of the genome wide association studies used in this study.

| **Traits** | **Data source (or PMID)** | **Inclusion Criteria for SNP** | **Imputation Quality Control**  **(r2/ INFO score)** | **Case Definition** | **Unit** | **Covariate adjustments** | **Imputation platfrom** |
| --- | --- | --- | --- | --- | --- | --- | --- |
| Heart failure | 31919418 | Study specific | Study specific INFO > 0.5 or 0.8 | Clinical Diagnosis of Heart Failure of any aetiology with no inclusion criteria based on LV ejection fraction | logodds | Sex, age (except for single-sex studies) and PCs for appropriate individual studies | 1KGP phase 1 or 3, Hapmap 2 NCBI build 36, Haplotype Reference Consortium, the Estonian WholeGenome Sequence reference, a reference sample based on 15,220 WGS of Icelandic individuals |
| Coronary heart disease | 26343387 | MAF >0.5% | r2 >0.3 or INFO >0.4 | inclusive CAD diagnosis (e.g. myocardial infarction, acute coronary syndrome, chronic stable angina, or coronary stenosis >50%) | logodds | Study-specific covariates and over-dispersion | 1KGP phase 1v3 |
| Atrial fibrillation | 30061737 | Study  specific | r2 > 0.3 | DiscovEGR participants with at least one electronic health record problem list entry or at least two diagnosis code entries for two separate clinical encounters on separate calendar days for ICD-10 I48: atrial fibrillation and flutter. | logodds | Sex , age, age^2^, first four PCs of ancestry | Haplotype Reference Consortium |
| Ischemic stroke | 29531354 | MAF ≥1% | INFO ≥ 0.5 | Defined as ischemic stroke or intracerebral hemorrhage based on clinical and imaging criteria | logodds | Study specific , Sex and Age as minimum | 1KGP phase 1v3 or similar |
| Hypertension | FinnGen | MAF >1% | info score > 0.6 | Persistently high systemic arterial blood pressure. Based on multiple readings (blood pressure determination), hypertension is currently defined as when systolic pressure is consistently greater than 140 mm Hg or when diastolic pressure is consistently 90 mm Hg or more. | logodds | sex, age, first 10 principal components, genotyping batch, and genetic relatedness | The 1000G Phase1 reference panels |

Table S2 Details of analyses used in this Mendelian randomization study.

| IVW | Inverse variance weighting regressed instrument-outcome associations on instrument-exposure associations for the instruments, weighted by the inverse of the variance of the instrument-outcome association, with the intercept constrained to 0. Inverse variance weighting gives valid causal estimate when all instruments are valid instruments, i.e. instruments related to exposures; instrument independent of confounders, and exclusion restriction assumption. Inverse variance weighting will produce valid causal estimate in the presence of balanced horizontal pleiotropy and the instrument strength independent of direct effect (InSIDE) assumption is satisfied. |
| --- | --- |
| MR-Egger | MR-Egger method is similar to inverse variance weighting, but the intercept is not constrained to 0. As such, the MR-intercept test evaluates whether there is presence of overall horizontal pleiotropy. This method gives valid causal estimate even when all instruments are invalid. However, it relies on InSIDE assumption, which could be violated if the instruments are related to the confounders of exposure-outcome association. |
| Weighted median | The weighted median method takes the weighted median of the Wald ratio as the causal estimate, weighted by the instrument-exposure association. Weighted median method gives valid causal estimate when more than 50% of the information is derived from valid instruments. |
| MR-PRESSO | MR-PRESSO provides a statistical test of (a) the presence of unbalanced horizontal pleiotropy (Global test), (b) generates a causal effect estimate corrected for unbalanced horizontal pleiotropy by outlier removal (Outlier corrected estimate), and (c) provides a statistical test comparing the estimate before and after the outlier removal (Distortion test). |

**Table S3 Mendelian randomization analysis of tea intake and atrial fibrillation.**

| **SNP** | **Chr** | **Position** | **A1** | **A2** | **EAF** | **Tea intake** | | | **Atrial fibrillation** | | |
| --- | --- | --- | --- | --- | --- | --- | --- | --- | --- | --- | --- |
|  |  |  |  |  |  | **Beta** | **SE** | ***P-*val** | **Beta** | **SE** | ***P-*val** |
| rs10752269 | 10 | 12692902 | A | G | 0.506 | -0.013 | 0.002 | 1.30E-09 | -0.002 | 0.007 | 0.716 |
| rs10764990 | 10 | 129152608 | A | G | 0.607 | -0.012 | 0.002 | 1.90E-08 | 0.005 | 0.007 | 0.498 |
| rs11164870 | 1 | 93552187 | G | C | 0.605 | -0.012 | 0.002 | 4.20E-08 | 0.001 | 0.007 | 0.887 |
| rs1156588 | 2 | 58515375 | G | A | 0.210 | -0.015 | 0.003 | 2.90E-09 | 0.003 | 0.008 | 0.744 |
| rs11587444 | 1 | 150722844 | G | A | 0.393 | 0.014 | 0.002 | 1.00E-10 | -0.006 | 0.007 | 0.404 |
| rs12591786 | 15 | 60902512 | T | C | 0.159 | -0.018 | 0.003 | 3.70E-10 | -0.004 | 0.009 | 0.707 |
| rs13282783 | 8 | 22088975 | T | C | 0.286 | -0.014 | 0.002 | 7.90E-09 | -0.010 | 0.008 | 0.188 |
| rs132904 | 22 | 41798896 | C | G | 0.779 | 0.017 | 0.003 | 7.80E-11 | -0.018 | 0.008 | 0.027 |
| rs141071726 | 7 | 17558580 | A | G | 0.027 | 0.041 | 0.007 | 2.20E-09 | 0.042 | 0.026 | 0.112 |
| rs1453548 | 11 | 59192089 | A | T | 0.665 | -0.013 | 0.002 | 3.00E-09 | -0.003 | 0.007 | 0.681 |
| rs1481012 | 4 | 89039082 | G | A | 0.112 | -0.026 | 0.003 | 5.30E-15 | -0.002 | 0.011 | 0.833 |
| rs149805207 | 6 | 137095269 | G | A | 0.009 | -0.072 | 0.013 | 1.10E-08 | -0.070 | 0.045 | 0.114 |
| rs17245213 | 11 | 1679769 | A | G | 0.208 | -0.015 | 0.003 | 2.00E-08 | 0.005 | 0.008 | 0.549 |
| rs17576658 | 13 | 100272019 | A | G | 0.247 | -0.013 | 0.002 | 4.10E-08 | -0.015 | 0.008 | 0.059 |
| rs17685 | 7 | 75616105 | A | G | 0.278 | 0.023 | 0.002 | 1.60E-22 | -0.002 | 0.007 | 0.811 |
| rs2273447 | 20 | 62900120 | T | A | 0.204 | 0.017 | 0.003 | 3.30E-11 | 0.002 | 0.009 | 0.811 |
| rs2279844 | 17 | 40819809 | A | G | 0.379 | -0.012 | 0.002 | 4.00E-08 | 0.001 | 0.007 | 0.893 |
| rs2351187 | 10 | 86850616 | A | G | 0.319 | 0.013 | 0.002 | 1.60E-08 | -0.002 | 0.007 | 0.808 |
| rs2472297 | 15 | 75027880 | T | C | 0.262 | 0.053 | 0.002 | 2.30E-109 | -0.006 | 0.008 | 0.426 |
| rs2478875 | 6 | 51283110 | G | A | 0.209 | 0.022 | 0.003 | 5.10E-17 | -0.016 | 0.008 | 0.048 |
| rs2645929 | 13 | 56444529 | G | A | 0.813 | -0.015 | 0.003 | 3.50E-08 | -0.015 | 0.009 | 0.072 |
| rs34619 | 5 | 60465365 | A | G | 0.431 | 0.012 | 0.002 | 4.30E-08 | 0.009 | 0.007 | 0.198 |
| rs4808193 | 19 | 19410622 | C | T | 0.335 | 0.015 | 0.002 | 1.70E-11 | -0.014 | 0.007 | 0.052 |
| rs4817505 | 21 | 34343828 | C | T | 0.390 | 0.015 | 0.002 | 4.20E-12 | -0.005 | 0.007 | 0.514 |
| rs56188862 | 1 | 174189269 | C | T | 0.387 | -0.016 | 0.002 | 4.30E-13 | 0.003 | 0.007 | 0.617 |
| rs56348300 | 9 | 7054124 | G | C | 0.185 | 0.016 | 0.003 | 6.10E-09 | 0.011 | 0.009 | 0.214 |
| rs57462170 | 3 | 50239803 | A | G | 0.109 | 0.019 | 0.003 | 1.90E-08 | 0.013 | 0.011 | 0.225 |
| rs57631352 | 19 | 4338173 | G | A | 0.297 | -0.013 | 0.002 | 1.70E-08 | -0.002 | 0.007 | 0.809 |
| rs6829 | 13 | 111531264 | T | C | 0.596 | -0.012 | 0.002 | 3.70E-08 | 0.006 | 0.007 | 0.354 |
| rs713598 | 7 | 141673345 | G | C | 0.402 | 0.013 | 0.002 | 5.20E-10 | 0.009 | 0.007 | 0.170 |
| rs72797284 | 5 | 152031650 | G | A | 0.271 | -0.017 | 0.002 | 7.00E-13 | 0.009 | 0.008 | 0.219 |
| rs7757102 | 6 | 137222671 | G | A | 0.555 | -0.012 | 0.002 | 3.10E-08 | 0.002 | 0.007 | 0.773 |
| rs9302428 | 16 | 24717600 | G | C | 0.636 | 0.012 | 0.002 | 2.60E-08 | 0.006 | 0.007 | 0.375 |
| rs9624470 | 22 | 24820268 | A | G | 0.580 | 0.025 | 0.002 | 1.30E-31 | 0.001 | 0.007 | 0.931 |
| rs9648476 | 7 | 39293033 | A | G | 0.623 | 0.013 | 0.002 | 1.10E-08 | 0.001 | 0.007 | 0.899 |
| rs977474 | 12 | 11284772 | T | C | 0.834 | 0.022 | 0.003 | 2.40E-14 | 0.001 | 0.010 | 0.883 |

Abbreviation: SNP, single nucleotide polymorphism; Chr, chromosome; EAF, effect allele frequency; SE, standard error;

Table S4 Mendelian randomization analysis of tea intake and coronary heart disease.

| **SNP** | **Chr** | **Position** | **A1** | **A2** | **EAF** | **Tea intake** | | | **Coronary heart disease** | | |
| --- | --- | --- | --- | --- | --- | --- | --- | --- | --- | --- | --- |
|  |  |  |  |  |  | **Beta** | **SE** | ***P-*val** | **Beta** | **SE** | ***P-*val** |
| rs10752269 | 10 | 12692902 | A | G | 0.506 | -0.013 | 0.002 | 1.30E-09 | 0.025 | 0.009 | 0.006 |
| rs10764990 | 10 | 129152608 | A | G | 0.607 | -0.012 | 0.002 | 1.90E-08 | 0.025 | 0.010 | 0.009 |
| rs11164870 | 1 | 93552187 | G | C | 0.605 | -0.012 | 0.002 | 4.20E-08 | -0.005 | 0.010 | 0.598 |
| rs1156588 | 2 | 58515375 | G | A | 0.210 | -0.015 | 0.003 | 2.90E-09 | -0.024 | 0.012 | 0.036 |
| rs11587444 | 1 | 150722844 | G | A | 0.393 | 0.014 | 0.002 | 1.00E-10 | -0.024 | 0.009 | 0.012 |
| rs12591786 | 15 | 60902512 | T | C | 0.159 | -0.018 | 0.003 | 3.70E-10 | 0.003 | 0.013 | 0.823 |
| rs13282783 | 8 | 22088975 | T | C | 0.286 | -0.014 | 0.002 | 7.90E-09 | 0.016 | 0.011 | 0.126 |
| rs132904 | 22 | 41798896 | C | G | 0.779 | 0.017 | 0.003 | 7.80E-11 | 0.005 | 0.011 | 0.655 |
| rs141071726 | 7 | 17558580 | A | G | 0.027 | 0.041 | 0.007 | 2.20E-09 | 0.053 | 0.049 | 0.279 |
| rs1453548 | 11 | 59192089 | A | T | 0.665 | -0.013 | 0.002 | 3.00E-09 | 0.001 | 0.010 | 0.951 |
| rs1481012 | 4 | 89039082 | G | A | 0.112 | -0.026 | 0.003 | 5.30E-15 | 0.023 | 0.015 | 0.135 |
| rs149805207 | 6 | 137095269 | G | A | 0.009 | -0.072 | 0.013 | 1.10E-08 | -0.110 | 0.074 | 0.137 |
| rs17245213 | 11 | 1679769 | A | G | 0.208 | -0.015 | 0.003 | 2.00E-08 | 0.012 | 0.012 | 0.310 |
| rs17576658 | 13 | 100272019 | A | G | 0.247 | -0.013 | 0.002 | 4.10E-08 | -0.004 | 0.012 | 0.745 |
| rs17685 | 7 | 75616105 | A | G | 0.278 | 0.023 | 0.002 | 1.60E-22 | 0.001 | 0.010 | 0.927 |
| rs2117137 | 3 | 89525505 | G | A | 0.405 | 0.013 | 0.002 | 1.70E-09 | 0.009 | 0.010 | 0.358 |
| rs2273447 | 20 | 62900120 | T | A | 0.204 | 0.017 | 0.003 | 3.30E-11 | 0.003 | 0.012 | 0.779 |
| rs2351187 | 10 | 86850616 | A | G | 0.319 | 0.013 | 0.002 | 1.60E-08 | 0.007 | 0.011 | 0.496 |
| rs2472297 | 15 | 75027880 | T | C | 0.262 | 0.053 | 0.002 | 2.30E-109 | -0.003 | 0.013 | 0.818 |
| rs2478875 | 6 | 51283110 | G | A | 0.209 | 0.022 | 0.003 | 5.10E-17 | -0.016 | 0.011 | 0.157 |
| rs2645929 | 13 | 56444529 | G | A | 0.813 | -0.015 | 0.003 | 3.50E-08 | -0.005 | 0.012 | 0.673 |
| rs34619 | 5 | 60465365 | A | G | 0.431 | 0.012 | 0.002 | 4.30E-08 | 0.000 | 0.009 | 0.978 |
| rs4808193 | 19 | 19410622 | C | T | 0.335 | 0.015 | 0.002 | 1.70E-11 | -0.007 | 0.010 | 0.501 |
| rs4817505 | 21 | 34343828 | C | T | 0.390 | 0.015 | 0.002 | 4.20E-12 | 0.005 | 0.010 | 0.617 |
| rs56188862 | 1 | 174189269 | C | T | 0.387 | -0.016 | 0.002 | 4.30E-13 | 0.001 | 0.009 | 0.927 |
| rs56348300 | 9 | 7054124 | G | C | 0.185 | 0.016 | 0.003 | 6.10E-09 | 0.011 | 0.011 | 0.337 |
| rs57462170 | 3 | 50239803 | A | G | 0.109 | 0.019 | 0.003 | 1.90E-08 | -0.020 | 0.015 | 0.190 |
| rs57631352 | 19 | 4338173 | G | A | 0.297 | -0.013 | 0.002 | 1.70E-08 | 0.007 | 0.011 | 0.504 |
| rs6829 | 13 | 111531264 | T | C | 0.596 | -0.012 | 0.002 | 3.70E-08 | -0.001 | 0.010 | 0.936 |
| rs713598 | 7 | 141673345 | G | C | 0.402 | 0.013 | 0.002 | 5.20E-10 | 0.018 | 0.010 | 0.080 |
| rs72797284 | 5 | 152031650 | G | A | 0.271 | -0.017 | 0.002 | 7.00E-13 | -0.004 | 0.011 | 0.694 |
| rs7757102 | 6 | 137222671 | G | A | 0.555 | -0.012 | 0.002 | 3.10E-08 | 0.005 | 0.009 | 0.609 |
| rs9302428 | 16 | 24717600 | G | C | 0.636 | 0.012 | 0.002 | 2.60E-08 | 0.010 | 0.010 | 0.297 |
| rs9624470 | 22 | 24820268 | A | G | 0.580 | 0.025 | 0.002 | 1.30E-31 | -0.016 | 0.010 | 0.098 |
| rs9648476 | 7 | 39293033 | A | G | 0.623 | 0.013 | 0.002 | 1.10E-08 | 0.002 | 0.010 | 0.829 |
| rs977474 | 12 | 11284772 | T | C | 0.834 | 0.022 | 0.003 | 2.40E-14 | -0.002 | 0.015 | 0.871 |

Abbreviation: SNP, single nucleotide polymorphism; Chr, chromosome; EAF, effect allele frequency; SE, standard error;

Table S5 Mendelian randomization analysis of tea intake and hypertension.

| **SNP** | **Chr** | **Position** | **A1** | **A2** | **EAF** | **Tea intake** | | | **Hypertension** | | |
| --- | --- | --- | --- | --- | --- | --- | --- | --- | --- | --- | --- |
|  |  |  |  |  |  | **Beta** | **SE** | ***P-*val** | **Beta** | **SE** | ***P-*val** |
| rs10752269 | 10 | 12692902 | A | G | 0.506 | -0.013 | 0.002 | 1.30E-09 | 0.006 | 0.010 | 0.549 |
| rs10764990 | 10 | 129152608 | A | G | 0.607 | -0.012 | 0.002 | 1.90E-08 | 0.015 | 0.009 | 0.111 |
| rs11164870 | 1 | 93552187 | G | C | 0.605 | -0.012 | 0.002 | 4.20E-08 | 0.005 | 0.009 | 0.616 |
| rs1156588 | 2 | 58515375 | G | A | 0.210 | -0.015 | 0.003 | 2.90E-09 | -0.006 | 0.011 | 0.587 |
| rs11587444 | 1 | 150722844 | G | A | 0.393 | 0.014 | 0.002 | 1.00E-10 | -0.007 | 0.009 | 0.488 |
| rs12591786 | 15 | 60902512 | T | C | 0.159 | -0.018 | 0.003 | 3.70E-10 | 0.007 | 0.015 | 0.646 |
| rs13282783 | 8 | 22088975 | T | C | 0.286 | -0.014 | 0.002 | 7.90E-09 | -0.016 | 0.009 | 0.093 |
| rs132904 | 22 | 41798896 | C | G | 0.779 | 0.017 | 0.003 | 7.80E-11 | -0.029 | 0.011 | 0.007 |
| rs141071726 | 7 | 17558580 | A | G | 0.027 | 0.041 | 0.007 | 2.20E-09 | -0.006 | 0.039 | 0.888 |
| rs1453548 | 11 | 59192089 | A | T | 0.665 | -0.013 | 0.002 | 3.00E-09 | 0.011 | 0.010 | 0.277 |
| rs1481012 | 4 | 89039082 | G | A | 0.112 | -0.026 | 0.003 | 5.30E-15 | -0.021 | 0.018 | 0.242 |
| rs149805207 | 6 | 137095269 | G | A | 0.009 | -0.072 | 0.013 | 1.10E-08 | -0.017 | 0.033 | 0.603 |
| rs17245213 | 11 | 1679769 | A | G | 0.208 | -0.015 | 0.003 | 2.00E-08 | 0.006 | 0.012 | 0.616 |
| rs17576658 | 13 | 100272019 | A | G | 0.247 | -0.013 | 0.002 | 4.10E-08 | 0.008 | 0.011 | 0.493 |
| rs17685 | 7 | 75616105 | A | G | 0.278 | 0.023 | 0.002 | 1.60E-22 | -0.006 | 0.009 | 0.553 |
| rs2273447 | 20 | 62900120 | T | A | 0.204 | 0.017 | 0.003 | 3.30E-11 | -0.012 | 0.013 | 0.380 |
| rs2279844 | 17 | 40819809 | A | G | 0.379 | -0.012 | 0.002 | 4.00E-08 | -0.010 | 0.010 | 0.296 |
| rs2351187 | 10 | 86850616 | A | G | 0.319 | 0.013 | 0.002 | 1.60E-08 | -0.008 | 0.010 | 0.414 |
| rs2472297 | 15 | 75027880 | T | C | 0.262 | 0.053 | 0.002 | 2.30E-109 | -0.020 | 0.011 | 0.058 |
| rs2478875 | 6 | 51283110 | G | A | 0.209 | 0.022 | 0.003 | 5.10E-17 | -0.018 | 0.012 | 0.145 |
| rs2645929 | 13 | 56444529 | G | A | 0.813 | -0.015 | 0.003 | 3.50E-08 | -0.001 | 0.013 | 0.919 |
| rs34619 | 5 | 60465365 | A | G | 0.431 | 0.012 | 0.002 | 4.30E-08 | -0.020 | 0.009 | 0.032 |
| rs4808193 | 19 | 19410622 | C | T | 0.335 | 0.015 | 0.002 | 1.70E-11 | 0.002 | 0.011 | 0.890 |
| rs4817505 | 21 | 34343828 | C | T | 0.390 | 0.015 | 0.002 | 4.20E-12 | -0.003 | 0.009 | 0.736 |
| rs56188862 | 1 | 174189269 | C | T | 0.387 | -0.016 | 0.002 | 4.30E-13 | 0.023 | 0.010 | 0.013 |
| rs56348300 | 9 | 7054124 | G | C | 0.185 | 0.016 | 0.003 | 6.10E-09 | 0.002 | 0.014 | 0.858 |
| rs57631352 | 19 | 4338173 | G | A | 0.297 | -0.013 | 0.002 | 1.70E-08 | 0.002 | 0.010 | 0.859 |
| rs6829 | 13 | 111531264 | T | C | 0.596 | -0.012 | 0.002 | 3.70E-08 | -0.005 | 0.009 | 0.604 |
| rs713598 | 7 | 141673345 | G | C | 0.402 | 0.013 | 0.002 | 5.20E-10 | 0.012 | 0.010 | 0.204 |
| rs72797284 | 5 | 152031650 | G | A | 0.271 | -0.017 | 0.002 | 7.00E-13 | 0.009 | 0.011 | 0.442 |
| rs7757102 | 6 | 137222671 | G | A | 0.555 | -0.012 | 0.002 | 3.10E-08 | 0.021 | 0.009 | 0.023 |
| rs9302428 | 16 | 24717600 | G | C | 0.636 | 0.012 | 0.002 | 2.60E-08 | 0.003 | 0.010 | 0.741 |
| rs9624470 | 22 | 24820268 | A | G | 0.580 | 0.025 | 0.002 | 1.30E-31 | 0.002 | 0.009 | 0.801 |
| rs9648476 | 7 | 39293033 | A | G | 0.623 | 0.013 | 0.002 | 1.10E-08 | -0.010 | 0.010 | 0.290 |
| rs977474 | 12 | 11284772 | T | C | 0.834 | 0.022 | 0.003 | 2.40E-14 | -0.001 | 0.016 | 0.966 |

Abbreviation: SNP, single nucleotide polymorphism; Chr, chromosome; EAF, effect allele frequency; SE, standard error;

Table S6 Mendelian randomization analysis of tea intake and heart failure.

| **SNP** | **Chr** | **Position** | **A1** | **A2** | **EAF** | **Tea intake** | | | **Heart failure** | | |
| --- | --- | --- | --- | --- | --- | --- | --- | --- | --- | --- | --- |
|  |  |  |  |  |  | **Beta** | **SE** | ***P-*val** | **Beta** | **SE** | ***P-*val** |
| rs10752269 | 10 | 12692902 | A | G | 0.506 | -0.013 | 0.002 | 1.30E-09 | -0.002 | 0.009 | 0.799 |
| rs10764990 | 10 | 129152608 | A | G | 0.607 | -0.012 | 0.002 | 1.90E-08 | 0.013 | 0.008 | 0.123 |
| rs1156588 | 2 | 58515375 | G | A | 0.210 | -0.015 | 0.003 | 2.90E-09 | 0.013 | 0.010 | 0.167 |
| rs11587444 | 1 | 150722844 | G | A | 0.393 | 0.014 | 0.002 | 1.00E-10 | 0.001 | 0.008 | 0.860 |
| rs12591786 | 15 | 60902512 | T | C | 0.159 | -0.018 | 0.003 | 3.70E-10 | 0.018 | 0.011 | 0.097 |
| rs13282783 | 8 | 22088975 | T | C | 0.286 | -0.014 | 0.002 | 7.90E-09 | 0.002 | 0.009 | 0.829 |
| rs1481012 | 4 | 89039082 | G | A | 0.112 | -0.026 | 0.003 | 5.30E-15 | -0.012 | 0.013 | 0.362 |
| rs17245213 | 11 | 1679769 | A | G | 0.208 | -0.015 | 0.003 | 2.00E-08 | -0.003 | 0.010 | 0.742 |
| rs17576658 | 13 | 100272019 | A | G | 0.247 | -0.013 | 0.002 | 4.10E-08 | 0.013 | 0.009 | 0.175 |
| rs17685 | 7 | 75616105 | A | G | 0.278 | 0.023 | 0.002 | 1.60E-22 | -0.001 | 0.009 | 0.882 |
| rs2117137 | 3 | 89525505 | G | A | 0.405 | 0.013 | 0.002 | 1.70E-09 | 0.001 | 0.008 | 0.926 |
| rs2279844 | 17 | 40819809 | A | G | 0.379 | -0.012 | 0.002 | 4.00E-08 | 0.007 | 0.008 | 0.373 |
| rs2351187 | 10 | 86850616 | A | G | 0.319 | 0.013 | 0.002 | 1.60E-08 | -0.008 | 0.008 | 0.319 |
| rs2478875 | 6 | 51283110 | G | A | 0.209 | 0.022 | 0.003 | 5.10E-17 | 0.002 | 0.010 | 0.826 |
| rs2645929 | 13 | 56444529 | G | A | 0.813 | -0.015 | 0.003 | 3.50E-08 | -0.006 | 0.010 | 0.562 |
| rs34619 | 5 | 60465365 | A | G | 0.431 | 0.012 | 0.002 | 4.30E-08 | -0.021 | 0.008 | 0.008 |
| rs4808193 | 19 | 19410622 | C | T | 0.335 | 0.015 | 0.002 | 1.70E-11 | -0.019 | 0.008 | 0.022 |
| rs4817505 | 21 | 34343828 | C | T | 0.390 | 0.015 | 0.002 | 4.20E-12 | -0.022 | 0.008 | 0.009 |
| rs56188862 | 1 | 174189269 | C | T | 0.387 | -0.016 | 0.002 | 4.30E-13 | 0.001 | 0.008 | 0.889 |
| rs57462170 | 3 | 50239803 | A | G | 0.109 | 0.019 | 0.003 | 1.90E-08 | -0.008 | 0.013 | 0.525 |
| rs57631352 | 19 | 4338173 | G | A | 0.297 | -0.013 | 0.002 | 1.70E-08 | -0.002 | 0.009 | 0.829 |
| rs6829 | 13 | 111531264 | T | C | 0.596 | -0.012 | 0.002 | 3.70E-08 | -0.001 | 0.008 | 0.907 |
| rs72797284 | 5 | 152031650 | G | A | 0.271 | -0.017 | 0.002 | 7.00E-13 | -0.013 | 0.009 | 0.147 |
| rs7757102 | 6 | 137222671 | G | A | 0.555 | -0.012 | 0.002 | 3.10E-08 | 0.003 | 0.008 | 0.718 |
| rs9624470 | 22 | 24820268 | A | G | 0.580 | 0.025 | 0.002 | 1.30E-31 | -0.004 | 0.008 | 0.619 |
| rs9648476 | 7 | 39293033 | A | G | 0.623 | 0.013 | 0.002 | 1.10E-08 | -0.012 | 0.008 | 0.155 |
| rs977474 | 12 | 11284772 | T | C | 0.834 | 0.022 | 0.003 | 2.40E-14 | -0.028 | 0.011 | 0.016 |

Abbreviation: SNP, single nucleotide polymorphism; Chr, chromosome; EAF, effect allele frequency; SE, standard error;

Table S7 Mendelian randomization analysis of tea intake and Ischemic stroke.

| **SNP** | **Chr** | **Position** | **A1** | **A2** | **EAF** | **Tea intake** | | | **Ischemic stroke** | | |
| --- | --- | --- | --- | --- | --- | --- | --- | --- | --- | --- | --- |
|  |  |  |  |  |  | **Beta** | **SE** | ***P-*val** | **Beta** | **SE** | ***P-*val** |
| rs10752269 | 10 | 12692902 | A | G | 0.506 | -0.013 | 0.002 | 1.30E-09 | 0.013 | 0.010 | 0.209 |
| rs10764990 | 10 | 129152608 | A | G | 0.607 | -0.012 | 0.002 | 1.90E-08 | 0.003 | 0.010 | 0.783 |
| rs11164870 | 1 | 93552187 | G | C | 0.605 | -0.012 | 0.002 | 4.20E-08 | 0.017 | 0.010 | 0.102 |
| rs1156588 | 2 | 58515375 | G | A | 0.210 | -0.015 | 0.003 | 2.90E-09 | -0.019 | 0.013 | 0.129 |
| rs11587444 | 1 | 150722844 | G | A | 0.393 | 0.014 | 0.002 | 1.00E-10 | -0.004 | 0.011 | 0.717 |
| rs12591786 | 15 | 60902512 | T | C | 0.159 | -0.018 | 0.003 | 3.70E-10 | 0.040 | 0.014 | 0.005 |
| rs13282783 | 8 | 22088975 | T | C | 0.286 | -0.014 | 0.002 | 7.90E-09 | 0.030 | 0.011 | 0.007 |
| rs132904 | 22 | 41798896 | C | G | 0.779 | 0.017 | 0.003 | 7.80E-11 | 5.00E-04 | 0.012 | 0.970 |
| rs141071726 | 7 | 17558580 | A | G | 0.027 | 0.041 | 0.007 | 2.20E-09 | -0.036 | 0.045 | 0.428 |
| rs1453548 | 11 | 59192089 | A | T | 0.665 | -0.013 | 0.002 | 3.00E-09 | -0.003 | 0.011 | 0.788 |
| rs1481012 | 4 | 89039082 | G | A | 0.112 | -0.026 | 0.003 | 5.30E-15 | 0.004 | 0.017 | 0.836 |
| rs149805207 | 6 | 137095269 | G | A | 0.009 | -0.072 | 0.013 | 1.10E-08 | 0.051 | 0.081 | 0.526 |
| rs17245213 | 11 | 1679769 | A | G | 0.208 | -0.015 | 0.003 | 2.00E-08 | -0.002 | 0.012 | 0.855 |
| rs17685 | 7 | 75616105 | A | G | 0.278 | 0.023 | 0.002 | 1.60E-22 | -0.002 | 0.012 | 0.861 |
| rs2117137 | 3 | 89525505 | G | A | 0.405 | 0.013 | 0.002 | 1.70E-09 | -0.004 | 0.010 | 0.680 |
| rs2273447 | 20 | 62900120 | T | A | 0.204 | 0.017 | 0.003 | 3.30E-11 | -0.022 | 0.015 | 0.136 |
| rs2279844 | 17 | 40819809 | A | G | 0.379 | -0.012 | 0.002 | 4.00E-08 | 0.010 | 0.010 | 0.320 |
| rs2351187 | 10 | 86850616 | A | G | 0.319 | 0.013 | 0.002 | 1.60E-08 | -0.009 | 0.011 | 0.407 |
| rs2478875 | 6 | 51283110 | G | A | 0.209 | 0.022 | 0.003 | 5.10E-17 | -0.006 | 0.012 | 0.615 |
| rs2645929 | 13 | 56444529 | G | A | 0.813 | -0.015 | 0.003 | 3.50E-08 | -0.002 | 0.013 | 0.870 |
| rs34619 | 5 | 60465365 | A | G | 0.431 | 0.012 | 0.002 | 4.30E-08 | -0.022 | 0.010 | 0.032 |
| rs4808193 | 19 | 19410622 | C | T | 0.335 | 0.015 | 0.002 | 1.70E-11 | -8.00E-04 | 0.011 | 0.936 |
| rs4817505 | 21 | 34343828 | C | T | 0.390 | 0.015 | 0.002 | 4.20E-12 | 0.006 | 0.010 | 0.560 |
| rs56188862 | 1 | 174189269 | C | T | 0.387 | -0.016 | 0.002 | 4.30E-13 | 0.010 | 0.010 | 0.309 |
| rs57462170 | 3 | 50239803 | A | G | 0.109 | 0.019 | 0.003 | 1.90E-08 | 0.009 | 0.017 | 0.610 |
| rs57631352 | 19 | 4338173 | G | A | 0.297 | -0.013 | 0.002 | 1.70E-08 | 0.017 | 0.011 | 0.130 |
| rs6829 | 13 | 111531264 | T | C | 0.596 | -0.012 | 0.002 | 3.70E-08 | -0.010 | 0.010 | 0.323 |
| rs713598 | 7 | 141673345 | G | C | 0.402 | 0.013 | 0.002 | 5.20E-10 | -0.007 | 0.011 | 0.511 |
| rs72797284 | 5 | 152031650 | G | A | 0.271 | -0.017 | 0.002 | 7.00E-13 | 0.020 | 0.011 | 0.070 |
| rs7757102 | 6 | 137222671 | G | A | 0.555 | -0.012 | 0.002 | 3.10E-08 | -0.014 | 0.010 | 0.159 |
| rs9302428 | 16 | 24717600 | G | C | 0.636 | 0.012 | 0.002 | 2.60E-08 | 5.00E-04 | 0.010 | 0.960 |
| rs9624470 | 22 | 24820268 | A | G | 0.580 | 0.025 | 0.002 | 1.30E-31 | -2.00E-04 | 0.010 | 0.984 |
| rs9648476 | 7 | 39293033 | A | G | 0.623 | 0.013 | 0.002 | 1.10E-08 | 0.004 | 0.010 | 0.720 |
| rs977474 | 12 | 11284772 | T | C | 0.834 | 0.022 | 0.003 | 2.40E-14 | -1.00E-04 | 0.016 | 0.995 |

Abbreviation: SNP, single nucleotide polymorphism; Chr, chromosome; EAF, effect allele frequency; SE, standard error

Table S8 Mendelian randomization analysis of hypertension and heart failure.

| **SNP** | **Chr** | **Position** | **A1** | **A2** | **EAF** | **Hypertension** | | | **Heart failure** | | |
| --- | --- | --- | --- | --- | --- | --- | --- | --- | --- | --- | --- |
|  |  |  |  |  |  | **Beta** | **SE** | ***P-*val** | **Beta** | **SE** | ***P-*val** |
| rs10059884 | 5 | 32832474 | A | C | 0.59 | 0.07 | 0.01 | 1.92E-12 | 0.01 | 0.008 | 0.24 |
| rs10185395 | 2 | 164447403 | T | G | 0.24 | 0.06 | 0.01 | 3.54E-08 | 0.00 | 0.011 | 0.88 |
| rs10206833 | 2 | 159462894 | A | G | 0.87 | -0.08 | 0.01 | 3.56E-09 | -0.01 | 0.014 | 0.30 |
| rs10217559 | 9 | 112751571 | T | C | 0.69 | 0.06 | 0.01 | 3.87E-09 | -0.01 | 0.008 | 0.53 |
| rs10757274 | 9 | 22096055 | G | A | 0.43 | 0.05 | 0.01 | 3.52E-08 | 0.06 | 0.008 | 0.00 |
| rs11636952 | 15 | 75114322 | C | T | 0.61 | -0.06 | 0.01 | 4.21E-09 | -0.01 | 0.009 | 0.46 |
| rs117515309 | 17 | 56404934 | A | C | 0.06 | -0.11 | 0.02 | 1.10E-08 | -0.03 | 0.023 | 0.26 |
| rs12509595 | 4 | 81182554 | C | T | 0.31 | 0.12 | 0.01 | 1.34E-31 | 0.02 | 0.009 | 0.02 |
| rs1275984 | 2 | 26911509 | C | A | 0.55 | -0.10 | 0.01 | 3.38E-25 | -0.02 | 0.008 | 0.06 |
| rs12828438 | 12 | 12883570 | G | A | 0.54 | -0.06 | 0.01 | 5.01E-11 | 0.00 | 0.008 | 0.94 |
| rs1317181 | 1 | 230873488 | T | G | 0.21 | 0.07 | 0.01 | 4.04E-10 | 0.00 | 0.009 | 0.90 |
| rs1374264 | 2 | 164999883 | C | A | 0.41 | -0.06 | 0.01 | 6.95E-11 | -0.01 | 0.008 | 0.08 |
| rs143439093 | 1 | 11981282 | G | A | 0.07 | -0.14 | 0.02 | 5.01E-15 | 0.02 | 0.028 | 0.42 |
| rs145153053 | 17 | 45138033 | G | A | 0.20 | 0.07 | 0.01 | 7.54E-10 | 0.03 | 0.011 | 0.00 |
| rs149150643 | 4 | 185877562 | C | T | 0.05 | -0.13 | 0.02 | 2.27E-09 | 0.01 | 0.018 | 0.74 |
| rs167479 | 19 | 11526765 | T | G | 0.42 | -0.08 | 0.01 | 2.66E-19 | -0.01 | 0.009 | 0.26 |
| rs16853076 | 3 | 168774754 | C | T | 0.06 | -0.11 | 0.02 | 1.06E-08 | -0.04 | 0.014 | 0.01 |
| rs1888693 | 10 | 18440444 | A | G | 0.39 | 0.06 | 0.01 | 1.45E-10 | 0.01 | 0.008 | 0.45 |
| rs2032451 | 6 | 26092170 | T | G | 0.11 | 0.09 | 0.01 | 1.28E-09 | 0.01 | 0.011 | 0.42 |
| rs2190994 | 7 | 150077617 | T | G | 0.28 | -0.06 | 0.01 | 5.09E-09 | -0.02 | 0.009 | 0.08 |
| rs2392929 | 7 | 106414069 | G | T | 0.30 | 0.06 | 0.01 | 5.21E-10 | 0.02 | 0.010 | 0.09 |
| rs2627313 | 15 | 81006712 | T | C | 0.40 | 0.05 | 0.01 | 7.35E-09 | 0.02 | 0.008 | 0.00 |
| rs2643826 | 3 | 27562988 | T | C | 0.40 | 0.06 | 0.01 | 1.75E-11 | 0.01 | 0.008 | 0.31 |
| rs2782981 | 10 | 115781547 | C | T | 0.65 | 0.08 | 0.01 | 8.71E-17 | 0.00 | 0.009 | 0.75 |
| rs28567725 | 16 | 53826028 | C | T | 0.41 | 0.06 | 0.01 | 2.76E-11 | 0.04 | 0.008 | 0.00 |
| rs303949 | 13 | 72365326 | C | A | 0.10 | 0.08 | 0.02 | 2.70E-08 | 0.00 | 0.013 | 0.90 |
| rs35427 | 12 | 115556307 | G | T | 0.36 | -0.06 | 0.01 | 5.91E-10 | -0.02 | 0.008 | 0.01 |
| rs3790604 | 1 | 113046879 | A | C | 0.17 | 0.13 | 0.01 | 1.40E-27 | 0.01 | 0.014 | 0.64 |
| rs3796585 | 4 | 156639174 | A | G | 0.35 | -0.08 | 0.01 | 4.48E-15 | 0.00 | 0.008 | 0.77 |
| rs3918226 | 7 | 150690176 | T | C | 0.07 | 0.17 | 0.02 | 7.08E-22 | 0.06 | 0.014 | 0.00 |
| rs4685218 | 3 | 14894140 | T | C | 0.10 | 0.10 | 0.02 | 1.30E-10 | 0.01 | 0.013 | 0.68 |
| rs4932373 | 15 | 91429287 | C | A | 0.27 | 0.08 | 0.01 | 5.58E-16 | 0.01 | 0.009 | 0.09 |
| rs557675 | 11 | 65566719 | G | T | 0.40 | -0.06 | 0.01 | 5.42E-11 | -0.02 | 0.008 | 0.00 |
| rs604723 | 11 | 100610546 | C | T | 0.74 | 0.06 | 0.01 | 1.36E-09 | 0.02 | 0.009 | 0.02 |
| rs62426324 | 6 | 127142458 | T | C | 0.50 | 0.05 | 0.01 | 7.13E-09 | 0.02 | 0.008 | 0.04 |
| rs62434119 | 6 | 150984431 | T | C | 0.08 | -0.13 | 0.02 | 6.23E-15 | -0.03 | 0.016 | 0.08 |
| rs62455829 | 7 | 70045931 | G | A | 0.28 | -0.06 | 0.01 | 5.68E-09 | 0.00 | 0.010 | 0.71 |
| rs629929 | 13 | 22318331 | T | G | 0.58 | 0.06 | 0.01 | 1.36E-10 | 0.01 | 0.008 | 0.46 |
| rs6668768 | 1 | 25136203 | T | C | 0.21 | 0.07 | 0.01 | 1.87E-09 | 0.01 | 0.012 | 0.24 |
| rs6860901 | 5 | 127871750 | T | C | 0.30 | 0.07 | 0.01 | 7.68E-12 | 0.02 | 0.009 | 0.00 |
| rs7134677 | 12 | 54441498 | T | C | 0.38 | -0.07 | 0.01 | 2.13E-15 | -0.01 | 0.009 | 0.12 |
| rs72779268 | 9 | 136454663 | A | C | 0.06 | -0.12 | 0.02 | 6.32E-11 | 0.01 | 0.020 | 0.80 |
| rs7483477 | 11 | 1920255 | G | T | 0.23 | 0.08 | 0.01 | 3.12E-12 | 0.01 | 0.009 | 0.18 |
| rs7545442 | 1 | 27260783 | T | C | 0.07 | 0.10 | 0.02 | 2.74E-08 | 0.03 | 0.014 | 0.08 |
| rs78412528 | 20 | 57734912 | A | G | 0.17 | 0.14 | 0.01 | 1.03E-30 | 0.03 | 0.012 | 0.03 |
| rs79873333 | 17 | 47357173 | T | C | 0.05 | -0.14 | 0.02 | 2.11E-10 | -0.01 | 0.025 | 0.76 |
| rs9899012 | 17 | 61545486 | A | G | 0.08 | -0.11 | 0.02 | 1.41E-10 | -0.03 | 0.018 | 0.07 |

Abbreviation: SNP, single nucleotide polymorphism; Chr, chromosome; EAF, effect allele frequency; SE, standard error

Table S9 Mendelian randomization analysis of hypertension and ischemic stroke.

| **SNP** | **Chr** | **Position** | **A1** | **A2** | **EAF** | **Hypertension** | | | **Ischemic stroke** | | |
| --- | --- | --- | --- | --- | --- | --- | --- | --- | --- | --- | --- |
|  |  |  |  |  |  | **Beta** | **SE** | ***P-*val** | **Beta** | **SE** | ***P-*val** |
| rs10059884 | 5 | 32832474 | A | C | 0.59 | 0.07 | 0.009 | 1.92E-12 | 0.017 | 0.010 | 0.10 |
| rs10185395 | 2 | 164447403 | T | G | 0.24 | 0.06 | 0.011 | 3.54E-08 | 0.020 | 0.014 | 0.15 |
| rs10206833 | 2 | 159462894 | A | G | 0.87 | -0.08 | 0.013 | 3.56E-09 | 0.018 | 0.018 | 0.32 |
| rs10217559 | 9 | 112751571 | T | C | 0.69 | 0.06 | 0.010 | 3.87E-09 | 0.021 | 0.011 | 0.05 |
| rs10757274 | 9 | 22096055 | G | A | 0.43 | 0.05 | 0.009 | 3.52E-08 | 0.049 | 0.010 | 0.00 |
| rs10786736 | 10 | 104849116 | C | G | 0.09 | -0.11 | 0.016 | 5.49E-12 | 0.001 | 0.016 | 0.95 |
| rs11636952 | 15 | 75114322 | C | T | 0.61 | -0.06 | 0.009 | 4.21E-09 | -0.029 | 0.011 | 0.01 |
| rs117515309 | 17 | 56404934 | A | C | 0.06 | -0.11 | 0.019 | 1.10E-08 | -0.032 | 0.029 | 0.27 |
| rs12509595 | 4 | 81182554 | C | T | 0.31 | 0.12 | 0.010 | 1.34E-31 | 0.022 | 0.011 | 0.04 |
| rs1275984 | 2 | 26911509 | C | A | 0.55 | -0.10 | 0.009 | 3.38E-25 | -0.020 | 0.011 | 0.07 |
| rs12828438 | 12 | 12883570 | G | A | 0.54 | -0.06 | 0.009 | 5.01E-11 | 0.005 | 0.010 | 0.62 |
| rs13112725 | 4 | 106911742 | C | G | 0.83 | 0.07 | 0.012 | 9.34E-09 | 0.023 | 0.012 | 0.06 |
| rs1317181 | 1 | 230873488 | T | G | 0.21 | 0.07 | 0.011 | 4.04E-10 | 0.003 | 0.012 | 0.81 |
| rs1358350 | 12 | 90101892 | A | T | 0.75 | 0.07 | 0.011 | 4.59E-10 | 0.028 | 0.011 | 0.01 |
| rs1374264 | 2 | 164999883 | C | A | 0.41 | -0.06 | 0.009 | 6.95E-11 | -0.001 | 0.010 | 0.94 |
| rs143439093 | 1 | 11981282 | G | A | 0.07 | -0.14 | 0.018 | 5.01E-15 | -0.009 | 0.037 | 0.82 |
| rs145153053 | 17 | 45138033 | G | A | 0.20 | 0.07 | 0.012 | 7.54E-10 | 0.045 | 0.013 | 0.00 |
| rs149150643 | 4 | 185877562 | C | T | 0.05 | -0.13 | 0.022 | 2.27E-09 | -0.023 | 0.023 | 0.32 |
| rs16853076 | 3 | 168774754 | C | T | 0.06 | -0.11 | 0.019 | 1.06E-08 | -0.054 | 0.018 | 0.00 |
| rs1888693 | 10 | 18440444 | A | G | 0.39 | 0.06 | 0.009 | 1.45E-10 | 0.001 | 0.011 | 0.94 |
| rs2032451 | 6 | 26092170 | T | G | 0.11 | 0.09 | 0.015 | 1.28E-09 | 0.036 | 0.014 | 0.01 |
| rs2190994 | 7 | 150077617 | T | G | 0.28 | -0.06 | 0.010 | 5.09E-09 | -0.001 | 0.011 | 0.94 |
| rs2392929 | 7 | 106414069 | G | T | 0.30 | 0.06 | 0.010 | 5.21E-10 | 0.035 | 0.012 | 0.00 |
| rs2627313 | 15 | 81006712 | T | C | 0.40 | 0.05 | 0.009 | 7.35E-09 | 0.029 | 0.010 | 0.00 |
| rs2643826 | 3 | 27562988 | T | C | 0.40 | 0.06 | 0.009 | 1.75E-11 | 0.019 | 0.010 | 0.06 |
| rs2782981 | 10 | 115781547 | C | T | 0.65 | 0.08 | 0.010 | 8.71E-17 | -0.010 | 0.011 | 0.38 |
| rs28567725 | 16 | 53826028 | C | T | 0.41 | 0.06 | 0.009 | 2.76E-11 | 0.011 | 0.010 | 0.28 |
| rs303949 | 13 | 72365326 | C | A | 0.10 | 0.08 | 0.015 | 2.70E-08 | 0.010 | 0.017 | 0.56 |
| rs34071855 | 1 | 10798489 | G | C | 0.42 | 0.10 | 0.009 | 1.04E-28 | 0.053 | 0.011 | 0.00 |
| rs35427 | 12 | 115556307 | G | T | 0.36 | -0.06 | 0.010 | 5.91E-10 | -0.038 | 0.011 | 0.00 |
| rs3790604 | 1 | 113046879 | A | C | 0.17 | 0.13 | 0.012 | 1.40E-27 | 0.067 | 0.018 | 0.00 |
| rs3796585 | 4 | 156639174 | A | G | 0.35 | -0.08 | 0.010 | 4.48E-15 | -0.015 | 0.010 | 0.13 |
| rs3918226 | 7 | 150690176 | T | C | 0.07 | 0.17 | 0.018 | 7.08E-22 | 0.061 | 0.019 | 0.00 |
| rs4685218 | 3 | 14894140 | T | C | 0.10 | 0.10 | 0.015 | 1.30E-10 | 0.009 | 0.017 | 0.58 |
| rs4932373 | 15 | 91429287 | C | A | 0.27 | 0.08 | 0.010 | 5.58E-16 | 0.046 | 0.011 | 0.00 |
| rs557675 | 11 | 65566719 | G | T | 0.40 | -0.06 | 0.009 | 5.42E-11 | -0.029 | 0.010 | 0.00 |
| rs604723 | 11 | 100610546 | C | T | 0.74 | 0.06 | 0.010 | 1.36E-09 | 0.025 | 0.011 | 0.03 |
| rs62426324 | 6 | 127142458 | T | C | 0.50 | 0.05 | 0.009 | 7.13E-09 | 0.026 | 0.010 | 0.01 |
| rs62434119 | 6 | 150984431 | T | C | 0.08 | -0.13 | 0.017 | 6.23E-15 | -0.029 | 0.020 | 0.15 |
| rs62455829 | 7 | 70045931 | G | A | 0.28 | -0.06 | 0.010 | 5.68E-09 | -0.015 | 0.013 | 0.25 |
| rs629929 | 13 | 22318331 | T | G | 0.58 | 0.06 | 0.009 | 1.36E-10 | 0.006 | 0.010 | 0.60 |
| rs6668768 | 1 | 25136203 | T | C | 0.21 | 0.07 | 0.011 | 1.87E-09 | 0.002 | 0.016 | 0.92 |
| rs6860901 | 5 | 127871750 | T | C | 0.30 | 0.07 | 0.010 | 7.68E-12 | 0.026 | 0.011 | 0.01 |
| rs6918791 | 6 | 126218961 | G | C | 0.73 | 0.06 | 0.010 | 2.15E-08 | 0.024 | 0.011 | 0.03 |
| rs6961048 | 7 | 27328187 | G | C | 0.14 | 0.07 | 0.013 | 3.39E-08 | 0.039 | 0.016 | 0.02 |
| rs7123467 | 11 | 10155174 | G | C | 0.20 | 0.07 | 0.011 | 2.47E-09 | 0.030 | 0.018 | 0.09 |
| rs7134677 | 12 | 54441498 | T | C | 0.38 | -0.07 | 0.009 | 2.13E-15 | -0.045 | 0.011 | 0.00 |
| rs72779268 | 9 | 136454663 | A | C | 0.06 | -0.12 | 0.019 | 6.32E-11 | -0.025 | 0.028 | 0.37 |
| rs7483477 | 11 | 1920255 | G | T | 0.23 | 0.08 | 0.011 | 3.12E-12 | 0.009 | 0.012 | 0.47 |
| rs7545442 | 1 | 27260783 | T | C | 0.07 | 0.10 | 0.018 | 2.74E-08 | 0.007 | 0.019 | 0.71 |
| rs78412528 | 20 | 57734912 | A | G | 0.17 | 0.14 | 0.012 | 1.03E-30 | 0.000 | 0.016 | 0.98 |
| rs79873333 | 17 | 47357173 | T | C | 0.05 | -0.14 | 0.022 | 2.11E-10 | 0.002 | 0.032 | 0.96 |
| rs9899012 | 17 | 61545486 | A | G | 0.08 | -0.11 | 0.017 | 1.41E-10 | -0.012 | 0.024 | 0.62 |

Abbreviation: SNP, single nucleotide polymorphism; Chr, chromosome; EAF, effect allele frequency; SE, standard error

Table S10 Statistical power analysis.

| **Exposure** | **Outcomes** | **Power (%)** |
| --- | --- | --- |
| Tea intake |  | 53 |
|  | Atrial fibrillation | 100 |
|  | Coronary heart disease | 100 |
|  | Hypertension | 100 |
|  | Heart failure | 100 |
|  | Ischemic stroke |  |
| Hypertension |  |  |
|  | Heart failure | 80 |
|  | Ischemic stroke | 98 |
